# Live substrate positively affects root growth and stolon direction in the woodland strawberry, *Fragaria vesca*

**DOI:** 10.3389/fpls.2015.00814

**Published:** 2015-09-30

**Authors:** Erica M. Waters, Maxine A. Watson

**Affiliations:** Department of Biology, Indiana University, Bloomington, INUSA

**Keywords:** plant nutrient foraging, clonal plants, woodland strawberry, *Fragaria vesca*, root biomass, stolon trajectory

## Abstract

Studies of clonal plant foraging generally focus on growth responses to patch quality once rooted. Here we explore the possibility of true plant foraging; the ability to detect and respond to patch resource status **prior to rooting**. Two greenhouse experiments were conducted to investigate the morphological changes that occur when individual daughter ramets of *Fragaria vesca* (woodland strawberry) were exposed to air above live (non-sterilized) or dead (sterilized) substrates. Contact between daughter ramets and substrate was prohibited. Daughter ramet root biomass was significantly larger over live versus dead substrate. Root:shoot ratio also increased over live substrate, a morphological response we interpret as indicative of active nutrient foraging. Daughter ramet root biomass was positively correlated with mother ramet size over live but not dead substrate. Given the choice between a live versus a dead substrate, primary stolons extended preferentially toward live substrates. We conclude that exposure to live substrate drives positive nutrient foraging responses in *F. vesca*. We propose that volatiles emitted from the substrates might be effecting the morphological changes that occur during true nutrient foraging.

## Introduction

Optimal foraging theory (OFT) proposes that organisms forage for nutrients in a way that maximizes energy intake per unit time (MacArthur and Pianka, 1966; Charnov, 1976; Norberg, 1977; Oaten, 1977). Resources often occur in patches within an environment and the theory predicts that there is an optimum pattern of visitation that provides an organism with maximum benefits for minimum output of energy. Application of the theory requires two conditions: (1) that individuals can move through and explore an environment and (2) that individuals can distinguish between and respond to patches of varying quality. The theory includes factors regarding both within-patch (“exploitation”, i.e., how long to remain, how to efficiently capture resources) and between-patch (“true foraging”, i.e., patterns of locating resources, time spent searching) behavior (Charnov, 1976; Oaten, 1977). Optimal adjustment of these factors results in an increased uptake of energy, and thus improves fitness.

Optimal foraging theory was originally used as a means of understanding and predicting animal behavior. It posited that animals adjust both foraging time and patch visitation order to maximize energy acquisition (MacArthur and Pianka, 1966; Charnov, 1976; Pyke et al., 1977; Pyke, 1984; McNamara et al., 2006). However, complex thought in animals involving predation risk (Brown, 1988; Higginson et al., 2012), food choice (Houston et al., 2011; Cressman et al., 2014), and memory (Freidin and Kacelnik, 2011) confounds the theory (Perry and Pianka, 1997).

**FIGURE 1:**
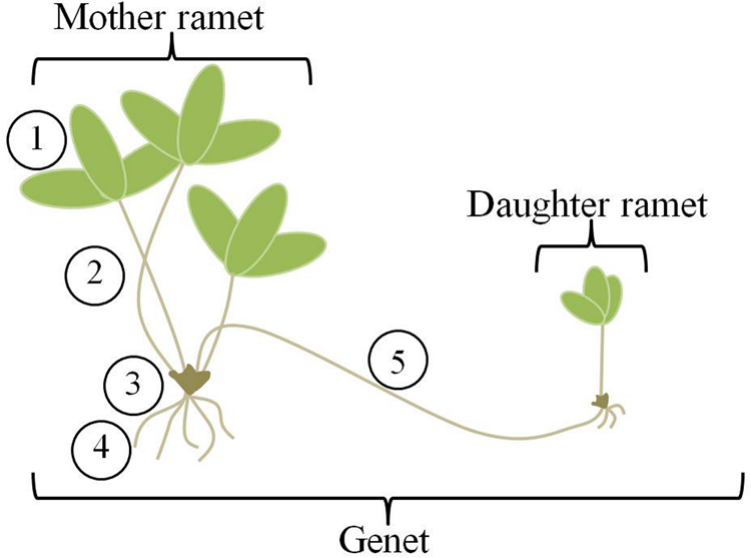
**Clone morphology.** Strawberry genets are made up of mother and daughter ramets connected by aboveground stolons. Individual mother and daughter ramets consist of five main organs (1) leaves, (2) petioles, (3) crown or stem, (4) roots, and (5) stolons. For analysis, we have combined the petioles and crown, labeling them “shoots”.

The theory has also been applied to clonal plants (Slade and Hutchings, 1987; Birch and Hutchings, 1994; Cain, 1994; de Kroon and Hutchings, 1995; Grime and Mackey, 2002). Clonal plants are unique in the plant world for the ability of genets (aggregates of plants that are the products of a single seed) to change their location over time, and therefore explore and effectively exploit a heterogeneous environment for light and nutrients. They do this through the production of ramets, or potentially physiologically independent genetically identical units (**Figure 1**). Compared to most animals, plant movement is slow; it occurs via growth processes and benefits accrue due to maintenance of connections between sister ramets. As new ramets are produced and extend into the environment, older ramets die, essentially moving the genet through space. Ramets remain connected via stolons or rhizomes for variable lengths of time (Jónsdóttir and Watson, 1997) and these connections allow for transport of nutrients and hormones between the mother and daughter ramets (Alpert and Mooney, 1986; Jónsdóttir and Watson, 1997; Hutchings, 1999). Thus, clonal plants fulfill the first requirement for application of OFT through their ability to move. But, can they do this in a selective way? Can they distinguish between and respond to patches of differing quality in a heterogeneous environment?

For plants to forage for light or nutrients, they must be able to sense, interpret, and respond to environmental signals that specify habitat quality in a way that results in the non-random placement of individual ramets in appropriate patches. Thus, clonal plant foraging can be said to occur if placement of daughter ramets occurs more frequently in high quality than in low quality patches (Cain, 1994). In stoloniferous plants (those with above-ground connections between ramets), the means of sensing and responding to light patches in a heterogeneous environment has been well studied (Slade and Hutchings, 1987; Methy et al., 1990; Kemball et al., 1992; Dong, 1993, 1995; Dong and Pierdominici, 1995; Stoll and Schmid, 1998; Grime and Mackey, 2002; Lepik et al., 2004; Dauzat et al., 2008). Detection of differences in red/far-red ratios via phytochromes and other photoreceptors induces plant morphological responses such as enhanced elongation rates or increased leaf area in response to low photosynthetically active radiation (PAR; Ballare et al., 1997; Smith, 2000; Franklin and Quail, 2010). These responses assist daughter ramets in locating and then occupying high light patches. Similarly, plants utilize red/far-red radiation ratios to assess areas of high vs. low density (i.e., neighbor sensing) (Schmitt and Wulff, 1993; Ballare, 1999; Marcuvitz and Turkington, 2000; Smith, 2000; Franklin and Quail, 2010). The majority of studies focused on morphological changes in leaf area and shoot biomass to differing light conditions, while others suggest that plasticity of spacers in length and branching intensity play a more critical role in light foraging, particularly in keeping ramets in light-rich patches (Kemball et al., 1992; Oborny, 1994; de Kroon and Hutchings, 1995; Dong, 1995; Dauzat et al., 2008).

Far less is known about the capacity of plants to detect nutrient-rich environments. Early studies focused on the proliferation of roots **after** plant establishment (“exploitation”) (Birch and Hutchings, 1994; Cain, 1994; de Kroon and Hutchings, 1995; van Kleunen and Fischer, 2001). Studies found that lateral root elongation is highly responsive to the presence of nitrates (Zhang and Forde, 2000), and results in an abundance of root mass in richer patches (Leyser and Fitter, 1998; Jansen et al., 2006). Connected ramets in complementary environments increase the size of organs that obtain the most abundant resource (Stuefer et al., 1996). In a light-rich environment clonal ramets increase the mass of shoots and leaves, whereas in a nutrient-rich environment, root growth is increased. While an overall increase in biomass indicates that ramets are situated in an abundance of resources, evidence of clonal ramet foraging arises when the ratio of root:shoot biomass increases or decreases in response to an increase in nutrients or light, respectively. These morphological changes indicate a preferential allocation of resources to specific organs specialized for the capture of the abundant resource (Tuomi et al., 1983). These studies mirror those related to light in that they indicate that once plants enter a rich environment they alter their morphology in ways that enhance exploitation. Evidence of between-patch foraging – the ability of a developing stolon to distinguish between nutrient-rich or nutrient-poor patches – also exists.

Precision of foraging depends on “the ability of a species to perceive the heterogeneity and respond to it” (Wijesinghe et al., 2001) and there is evidence of this ability in many clonal plants. Salzman (1985) demonstrated that when given a choice between saline or non-saline soils, *Ambrosia psilocstachya* placed 67% of its rhizomes in non-saline soils. While it may be argued that the salinity suppressed plant growth, similar patterns of nutrient patch detection also have been found in stoloniferous plants. *Cuscuta subinclusa* exhibited coiling responses prior to physiological connection and exploitation of its host, indicating an ability to survey and interpret its surroundings and adjust development appropriately (Kelly, 1990). To date, the most striking example of patch recognition and differentiation was reported by Roiloa and Retuerto (2006). They found that offspring ramets of *Fragaria vesca*, when given a choice of six soils of varying quality, preferentially grew into higher quality soils first. Only after these higher quality soils were colonized did the newest formed ramets colonize lower quality patches. These findings were in stark contrast to the homogeneous control, where daughter ramet placement was random. This experiment was particularly interesting because unlike *A. psilocstachya*, *F. vesca* is stoloniferous, demonstrating that clonal plants are capable of precision foraging aboveground. *While the study demonstrated that F. vesca are able to detect and respond to nutrient-rich patches, it did not investigate the morphological changes that occur when the ramet encounters a nutrient-rich patch, and raises the question: are there changes and, if so, are they consistent with optimal nutrient foraging?*

We conducted a series of experiments designed to investigate the morphological changes that occur in developing ramets ***prior to rooting*** in response to unsterilized (live) versus sterilized (dead) field substrate. Our goal was to determine whether air-borne signals are able to alter development such that newly developing ramets can be placed into favorable nutrient patches.

First, we examined local root growth and development of *F. vesca* daughter ramets exposed to live versus dead, nutrient-rich field substrates. Once a ramet roots, it can no longer move, so if true foraging is to occur, there must be air-borne clues signaling soil quality. Therefore, during the experiments, rooting into the substrate was prevented. We hypothesized that **unrooted** ramets exposed to air above live substrates would exhibit an increase in root biomass and root:shoot ratio compared to dead controls.

Because prior studies demonstrated that ramets of *F. vesca* are placed into higher quality soils first (Roiloa and Retuerto, 2006), we also developed two experiments to look at the trajectory of stolon extension when given the choice of nutrient-rich versus nutrient-poor patches. We hypothesized that there will be a positive response in the direction of growth of the extending stolon, such that it grows toward the rich substrate. Both parameters, a positive alteration of stolon direction and an increase in the root biomass and root:shoot ratio of **unrooted** ramets, would be taken as a positive indicators of nutrient foraging.

## Materials and Methods

### Experimental Species

*Fragaria vesca*, the woodland strawberry, is an herbaceous perennial native to the northern hemisphere. Growth occurs clonally via production of stolons; distribution of ramets within a colony indicates a guerrilla growth form (Angevine, 1983).

### Substrate Collection

In order to determine responsiveness to airborne signals from live substrate, we collected soil and litter from a site colonized by *F. vesca* in Aurora, IN (N 39 05.225 W 084 55.663) in March and September 2012. Prior to collection, all strawberries were removed from the substrate. Leaf and stick litter was harvested and placed in plastic bags. Field soil no more than three inches deep was collected and stored in plastic bags. Any residual root mass in the soil was removed prior to storage. Soil and litter were stored in cool, dry, dark conditions until the experiment was initiated. At the beginning of each experiment, half of the collected field soil and litter was autoclaved, the other half was not.

### *Fragaria Vesca* Propagation

Woodland strawberries were propagated from a single clone in the greenhouse at Indiana University, Bloomington, IN, USA in the spring of 2012, the fall of 2012, the fall of 2013 and again in the spring of 2014. These genetically identical ramets were individually potted in 12 cm diam. pots filled with SunGro Metro-Mix and watered daily.

### Root Growth Experimental Design and Plant Data Collection

**FIGURE 2:**
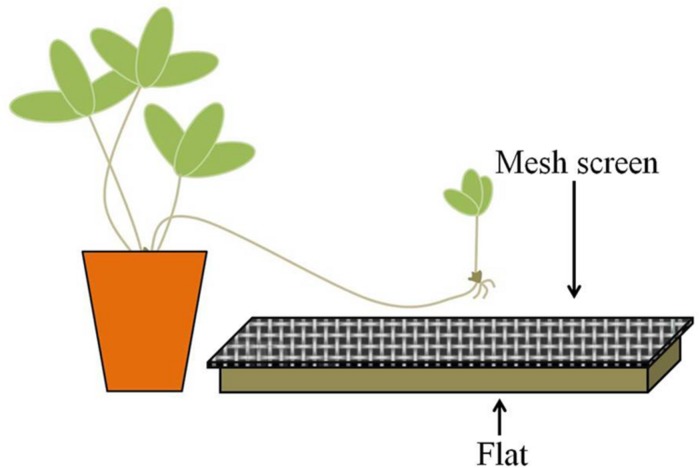
**Experimental scheme.** Strawberry daughter ramets were allowed to grow over flats filled with non-sterilized (live) or sterilized (dead) substrates. Mesh screens were placed on top of the flats, approximately two centimeters above the substrate, to prevent contact between the developing ramet and the substrate.

In the spring of 2012, 15 flats (27.3 cm × 54.0 cm × 6.1 cm) each were filled with 1L of field soil and covered with 0.5 L of leaf litter; an additional 15 contained dead (autoclave-sterilized) field substrate (soil plus litter). In order to prevent direct contact between developing ramets and the substrate, a sheet of aluminum screen mesh was placed over each flat; the mesh was situated approximately two centimeters above the substrate (**Figure 2**). Treatments were randomly placed along both sides of a bench in the greenhouse so that half faced east and half faced west.

Thirty potted strawberries with new daughter stolons at least 35 cm in length were randomly assigned to a treatment (live or dead) and were placed at the short edge of each flat, one per flat. The daughter stolon was directed toward and allowed to extend over the mesh-covered substrate. Developing stolons and ramets were not allowed to root into or come into direct contact with the substrate. Both the mother plants and substrate flats were watered daily with tap water and subjected to a 16-h light cycle. The identical experiment was repeated in the fall of 2012 with 80 individual ramets of the same genotype.

Primary stolon length was measured daily. All other newly emerging stolons were clipped from the mother over the duration of the experiment. Dates of initiation of daughter ramet development and root formation were recorded. Initiation of ramet development was identified by the upward curling of the stolon tip, accompanied by leaf production. Root formation was defined by the presence of primordial root hairs extending from the base of the developing daughter ramet. Three days after root formation on the daughter ramet, the entire assemblage (mother and daughter ramet) was removed from the experiment, harvested and separated into organs; for each ramet, the stem and petioles were combined and labeled “shoot” (**Figure 1**). Leaves were scanned into tif files and Image J (U.S. National Institutes of Health, Bethesda, MD, USA) was used to measure leaf area. All plant organs, including leaves were dried at 60°C for 3 days and weighed to mean ± 1 mg.

### Statistical Analysis

The statistical analyses were designed to determine if there was an effect of substrate type (live versus dead) on organs of the daughter ramet. Because previous studies indicated that the size of the mother ramet can affect growth responses (e.g., Cain, 1990), we also examined the effect of mother ramet size. Data sets were analyzed for normality based on QQ plots and Kolmogorov–Smirnov test values: for the spring 2012 data, all factors were log transformed to establish normality; for the fall 2012 data only daughter ramet aboveground dry mass, daughter ramet leaf dry mass, daughter ramet root dry mass and stolon length were log transformed, while daughter shoot dry mass, stolon dry mass and total mother dry mass were not. We performed a series of ANCOVAS on the daughter ramet leaf dry mass, daughter ramet shoot dry mass (later combined as aboveground dry mass), daughter ramet root dry mass, stolon dry mass and stolon length. Mother ramet total dry mass (maternal effect) was analyzed as a covariate for all factors. We used partial Eta squared (η^2^) to estimate the effect size. All analyses were performed using SPSS (IBM Corp., Released 2011. IBM SPSS Statistics for Windows, Version 20.0. Armonk, NY: IBM Corp.,).

### Stolon Trajectory Experimental Design and Plant Data Collection

**FIGURE 3:**
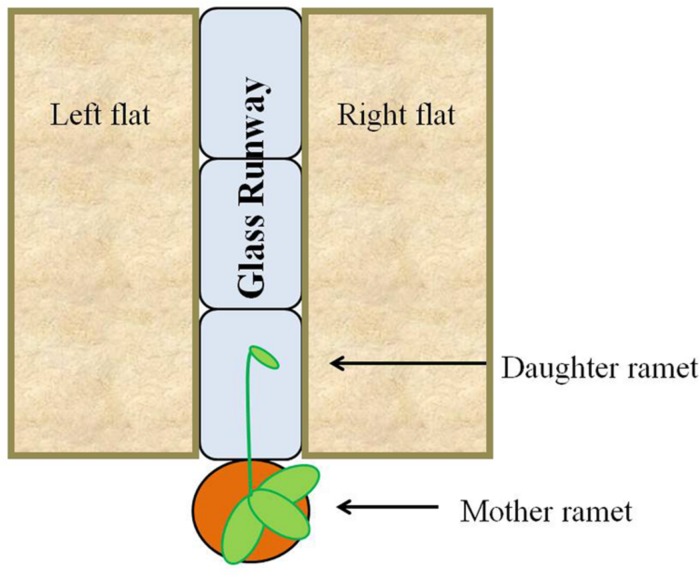
**Experimental scheme.** Strawberry daughter ramets were allowed to grow over a glass runway flanked with non-sterilized (live) or sand/sterilized (dead) substrates. Distribution of live and dead substrate on the left or right side of the plant was randomized.

In the fall of 2013, forty-three flats (27.3 cm × 54.0 cm × 6.1 cm) each were filled with 1 L of live field soil and covered with 0.5 L of leaf litter (live substrate). An additional 43 flats (27.3 cm × 54.0 cm × 6.1 cm) each were filled with autoclaved play sand (Hardscapes by Quikrete). Treatments consisted of a runway made of three glass blocks (6 in. × 8 in. × 4 in., Pittsburgh Corning Premiere). Each runway was flanked by one flat filled with live substrate and one flat filled with sand; distribution of live substrate on the left versus right side was randomized (**Figure 3**). Treatments were randomly placed along both sides of a bench in the greenhouse so that approximately half faced east and half faced west. Forty-three potted strawberries with new daughter stolons at least 35 cm in length were randomly assigned to treatments and placed at the short edge of the runway, one per runway. The daughter stolon was directed toward and allowed to extend along the glass runway. Both mother plants and substrate flats were watered daily with tap water and subjected to a 16-h light cycle. The identical experiment was repeated in the spring of 2014 with the following changes: (1) there were 46 individual strawberries (23 per treatment) and (2) the sand treatment was replaced with sterilized field substrate and litter (dead substrate).

Primary stolon growth was monitored daily. Once a developing stolon extended beyond the edge of the glass blocks, either in the direction of the live or the dead substrate, or off the end of the runway, the date was recorded and the individual was removed from the experiment. All other newly developing stolons were clipped from the mother over the duration of the experiment.

### Statistical Analysis

These statistical analyses were designed to determine if there was an effect of substrate type (live versus dead/sand) on the direction of stolon extension. Because there was an equal probability of the strawberry stolon extending into the live substrate, the dead substrate, or growing past the end of the glass runway (no choice), we performed a series of chi-square analyses with the final choice as the categorical variable. All analyses were performed using SPSS (IBM Corp., Released 2011. IBM SPSS Statistics for Windows, Version 20.0. Armonk, NY: IBM Corp.,).

## Results

### Root Experiment: Spring (**Table 1**)

Exposure to live substrate affected individual plant organs to different degrees. Most notably, average root dry mass was nearly three times greater on daughter ramets exposed to live versus dead substrates (*p* < 0.001). There was no significant difference in size of leaves, shoots or stolons in plants growing over live versus dead substrate. Because we considered changes in the root:shoot ratio as indicative of nutrient foraging, we analyzed the difference in root:shoot ratio between ramets exposed to the two substrate treatments. We found a significantly higher root:shoot ratio over live (0.02) versus dead substrate (0.01) [*F*(1,28) = 42.56, *p* ≪ 0.001).

**FIGURE 4:**
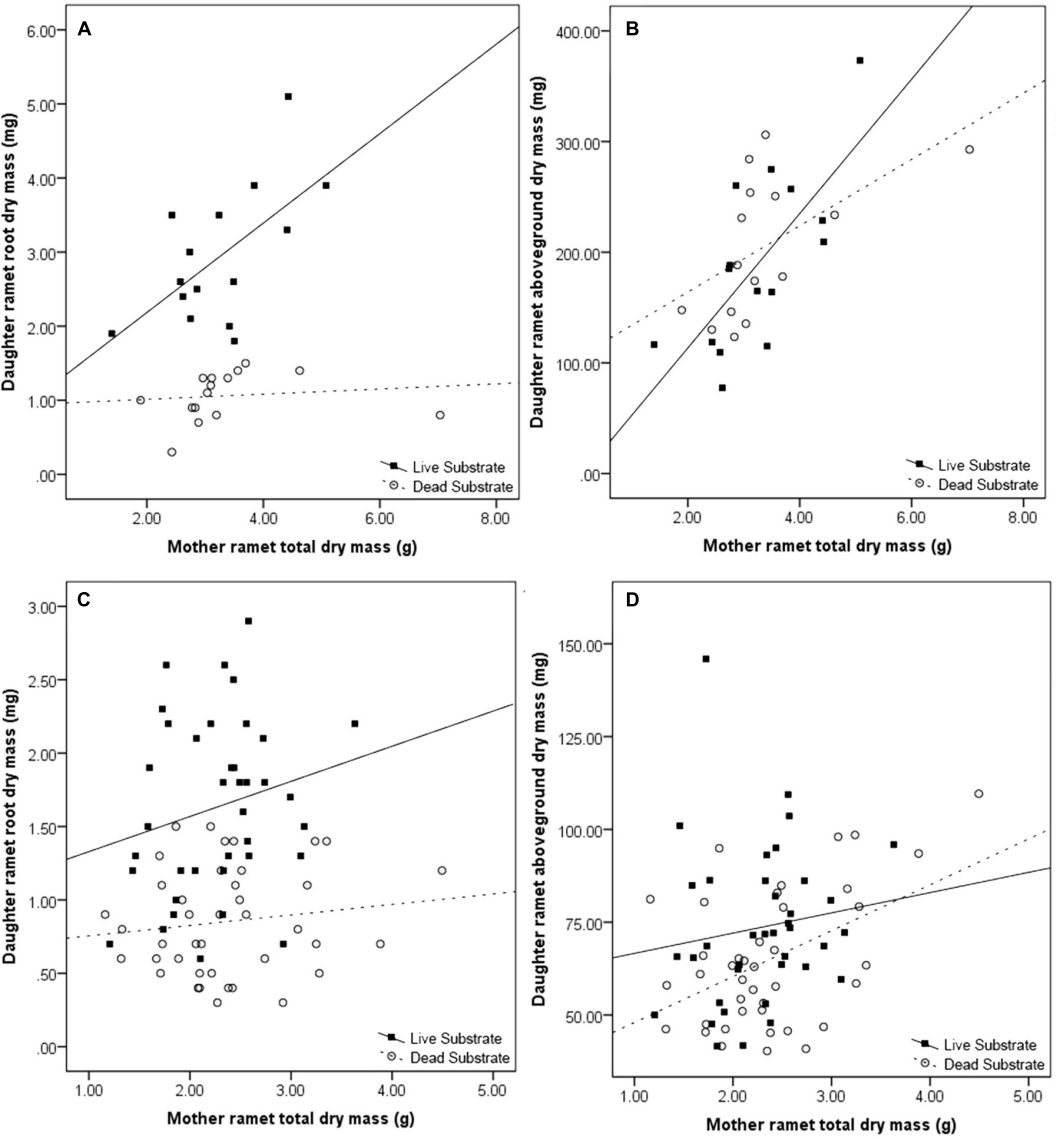
**Effects of maternal ramet size on daughter ramet root dry mass **(A,C)** and daughter ramet aboveground dry mass **(B,D)** in the spring **(A,B)** and fall **(C,D)**.** Filled boxes (■) represent ramets exposed to live substrate and open circles (○) represent ramets exposed to dead substrates.

Maternal effect differed between substrate treatments. Daughter ramet root biomass over dead substrate was independent of maternal size, whereas over live substrate, there was a strong correlation between the two (*r*^2^ = 0.561; *n* = 15; *p* = 0.015; **Figure 4A**) In contrast, aboveground dry mass (leaf + shoot) was significantly correlated with mother ramet size over both live (*r*^2^ = 0.656, *n* = 15; *p* = 0.004) and dead (*r*^2^ = 0.599; *n* = 15; *p =* 0.009) substrate*s* (**Figure 4B**).

### Root Experiment: Fall (**Table 2**)

Similar to the results from the spring experiment, root dry mass was significantly greater when daughter ramets were exposed to live substrate (*p* < 0.001). Substrate treatment had a significant effect on shoot dry mass (*p* = 0.03). Leaf dry mass was only marginally affected (*p* = 0.06) although daughter ramets produced more over live than dead substrate. Neither stolon dry mass nor stolon length was affected by substrate treatment. These results differ from those obtained in the spring, when only root dry mass was significantly affected by substrate type. As before, root:shoot ratio was significantly higher in daughter ramets exposed to live (0.02) versus dead substrate (0.01) [*F*(1,76) = 29.74, *p* ≪ 0.001]. Interestingly, the root:shoot ratios were similar between spring and fall.

Consistent with results from the spring experiment, daughter ramet root biomass over dead substrate was independent of maternal size, while over live soils the two factors were marginally correlated (*r*^2^ = 0.242; *n* = 38; *p =* 0.072; **Figure 4C**). Mother ramet size was significantly correlated with aboveground dry mass over dead (*r*^2^ = 0.179; *n* = 40; *p =* 0.003) but not live substrate (**Figure 4D**).

### Stolon Trajectory Experiment

In order to rule out any developmentally predetermined directional growth of the stolon, we analyzed the frequency of the stolon extending to the left versus the right of the glass blocks and found no statistically significant preference for growth direction (χ^2^ = 2.78; df = 1; *p* = 0.096).

**FIGURE 5:**
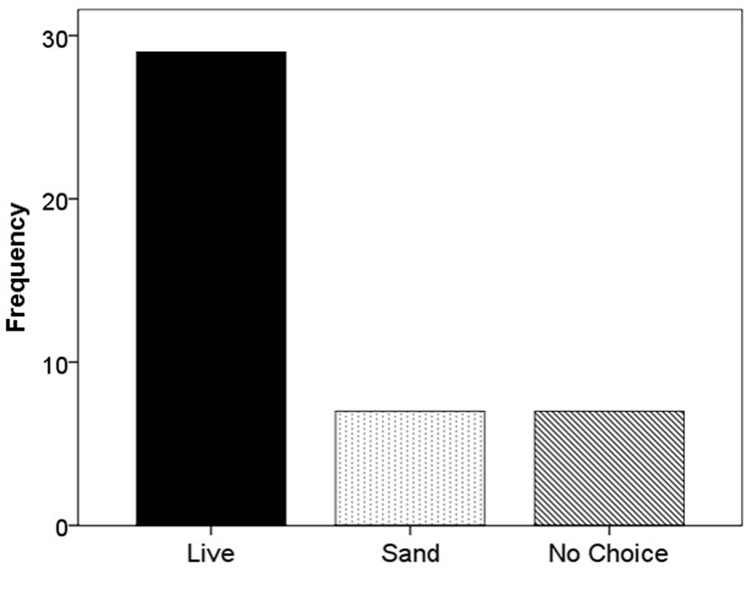
**Frequency of selection of extending stolons between live substrate (■), sand (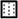), or no choice (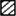)**.

**FIGURE 6:**
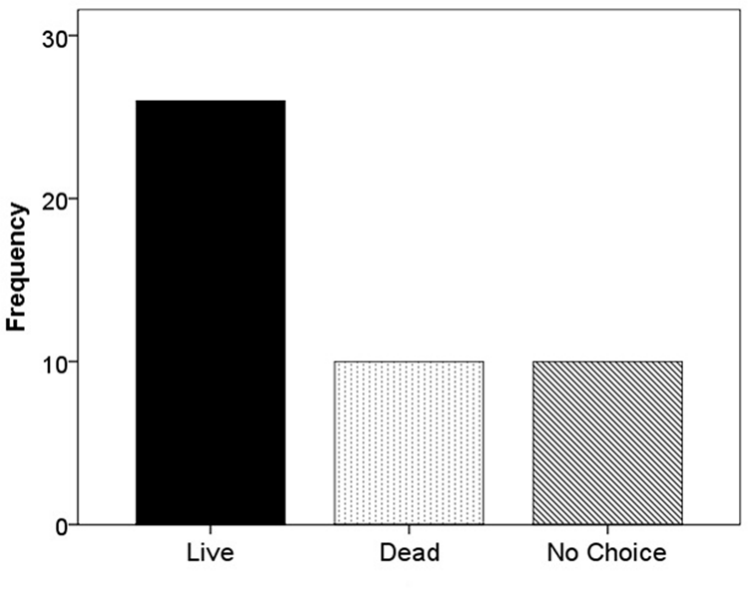
**Frequency of selection of extending stolons between live substrate (■), dead substrate (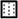), or no choice (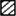)**.

When *F. vesca* stolons were given the choice of live substrate or sand, 67.4% grew into the live substrate, 16.3% grew into the sand, and 16.3% extended beyond the glass runway (χ^2^ = 22.52; df = 2; *p* < 0.001; **Figure 5**). When the experiment was repeated using live versus dead substrate, 56.5% grew into live substrate, 21.7% grew into dead substrate, and 21.7% extended beyond the glass runway (χ^2^ = 11.13; df = 2; *p* < 0.05; **Figure 6**).

## Discussion

We hypothesized that in order to grow into nutrient rich patches, the developing daughter ramet must be able to: (1) detect the patch and (2) respond to the presence of the patch. We suggest two types of responses indicative of nutrient foraging: (1) an increase in root biomass and root:shoot ratio prior to rooting and (2) an alteration of stolon growth trajectory in the direction of a nutrient rich patch. As a first step in testing this hypothesis, we asked whether root growth differs when a ramet extends over live versus dead substrate.

We found a consistent effect of substrate (live versus dead) on the daughter ramet root dry mass; daughter ramets produced more root biomass over live substrate (**Tables 1** and **2**). We also saw an increase in root:shoot ratio, an indicator of the relative allocation of biomass. Not only were our results consistent with our hypothesis, but also ratios in the spring and fall for both treatments were nearly identical. Because ramets were not allowed to come into contact with the substrate, this suggests that the consistency in root:shoot ratio is a programmed response of the daughter ramet to the presence of volatiles emitted from the substrate. The increase in root biomass correlated with maternal size over live (but not dead) substrate (**Figure 3**). One possible explanation for this pattern is that exposure to live substrates initiates a cascade of events increasing the distribution of resources from mother to daughter ramet.

**Table 1:** Analysis from the spring experiment.

	Mean		Treatment effect		Maternal effect		
Factor	Live substrate treatment	Dead substrate treatment	*F*(1,27)	*P*	*F*(1,27)	*P*	Effect size (η^2^)
Leaf dry mass (mg)	139.56 ± 15.32	150.40 ± 12.59	0.55	0.46	12.82	**^2^0.001**	0.32
Shoot dry mass (mg)	49.88 ± 5.27	54.64 ± 4.07	1.03	0.32	22.39	**<0.001**	0.45
Root dry mass (mg)	2.94 ± 0.24	1.06 ± 0.08	68.05	**<0.001**	2.93	0.10	0.10
Stolon dry mass (mg)	218.73 ± 28.44	242.69 ± 32.16	0.17	0.69	28.95	**<0.001**	0.52
Stolon length (mm)	296.47 ± 16.23	302.53 ± 20.47	0.00	0.96	8.05	**0.01**	0.23


*Effects of live versus dead substrate (treatment effect) and total dry mass of the mother ramet (maternal effect) on the organs of the daughter ramet. Bold face highlights effects significant at *p* < 0.05.*

**Table 2:** Analysis from the fall experiment.

	Mean		Treatment effect		Maternal effect		
Factor	Live substrate treatment	Dead substrate treatment	*F*(1,75)	*P*	*F*(1,75)	*P*	Effect size (η^2^)
Leaf dry mass (mg)	49.79 ± 2.72	43.75 ± 2.12	3.56	0.06	6.22	**0.01**	0.08
Shoot dry mass (mg)	23.77 ± 0.94	21.15 ± 0.91	4.70	**0.03**	5.04	**0.03**	0.06
Root dry mass (mg)	1.63 ± 0.09	0.85 ± 0.06	48.74	**<0.001**	1.96	0.17	0.03
Stolon dry mass (mg)	285.56 ± 10.67	267.75 ± 11.22	2.37	0.13	21.97	**<0.001**	0.28
Stolon length (mm)	469.18 ± 11.87	464.43 ± 12.59	0.19	0.66	1.99	0.16	0.03

*Effects of live versus dead substrate (treatment effect) and total dry mass of the mother ramet (maternal effect) on the organs of the daughter ramet. Bold face highlights effects significant at *p* < 0.05.*

In terms of stolon trajectory, we expected to see a higher frequency of growth toward nutrient rich versus nutrient poor patches. In both experiments, our results were consistent with our hypothesis, in that the majority of *F. vesca* individuals extended stolons into the live substrate (**Figures 4** and **5**). Furthermore, in both experiments, the frequency of stolon extension into nutrient-poor flats (sand or dead substrate) occurred equally, suggesting no differential influence from the less ideal patch.

The experiments in this study strongly demonstrate that ramets of *F. vesca* can identify and respond to nutrient-rich substrate patches, however, the mechanisms behind this capacity are less clear. Because the ability of a new ramet to explore an environment ends once rooting occurs, it is fundamentally important for the plant to be able to predict (based on environmental cues) the quality of the surrounding substrate. Thus, we designed our experiments in such a way to highlight morphological responses to nutrient-rich and nutrient-poor substrates independent of soil contact. Our positive results, specifically the increase in root:shoot ratio of developing ramets and the alteration of stolon trajectory, have led us to propose the following mechanism of patch detection.

The soil environment is highly heterogeneous, and the nature of the soil environment is primarily determined by its inhabitants; a nutrient-rich environment also is a substrate environment rich in microflora, microinvertebrates or larger fauna (Chaparro et al., 2012). Belowground volatile emission can influence the community (Wenke et al., 2010; Tumlinson, 2014) by controlling the bacterial and fungal population (Fiddaman and Rossall, 1993; Mackie and Wheatley, 1999; Kai et al., 2007; Vespermann et al., 2007), attracting herbivores (Neveu et al., 2002; Rasmann et al., 2005; Johnson and Gregory, 2006; Ali et al., 2010), and moderating plant growth (Ryu et al., 2003; Splivallo et al., 2007). It is highly likely that the soil inhabitants produce volatile organic compounds (VOCs) that could be detected by a foraging clonal plant. In this scenario, a developing ramet at the terminal end of an extending stolon would have an opportunity to effectively sample the nutrient environment without the morphological commitment to rooting. This would increase the likelihood of the plant establishing roots in a nutrient-rich/high-quality patch and would explain how a stoloniferous clonal plant might identify and grow into these patches.

Volatile organic compounds are naturally produced chemicals that are critical in influencing ecological interactions both above and belowground (Hughes and Sperandio, 2008; Faure et al., 2009; Kai et al., 2009; Insam and Seewald, 2010; Wenke et al., 2010; Tumlinson, 2014). They are produced by a large variety of organisms, including microbes (Zhang et al., 2007; Kai et al., 2009; Ortiz-Castro et al., 2009), fungi (Splivallo et al., 2007; Tarkka and Piechulla, 2007; Morath et al., 2012; Hung et al., 2015), and plants (Niinemets et al., 2004; Kant et al., 2009; Zhao et al., 2011). Along with mediating communication between different species, they also are byproducts released in response to temperature changes (Asensio et al., 2007; Zhao et al., 2011; Hartikainen et al., 2012), herbivory (Farmer, 2001; Rasmann et al., 2005; Poelman et al., 2013), pathogens (Huang et al., 2012; Panka et al., 2013), and drought (Asensio et al., 2012; Bourtsoukidis et al., 2014; Copolovici et al., 2014).

Volatile organic compounds are often implicated in the promotion of secondary responses, including plant growth. One highly cited example demonstrated that compounds emitted from *Bacilus subtilis* GB03 and *B. amyloliquefaciens* IN937a significantly increased the growth of *Arabidopsis* seedlings as compared to a non-growth promoting strain of *E. coli* and water controls (Ryu et al., 2003). In a similar study, Kai and Piechulla (2009) looked at the effects of *Serratia odorifera* volatiles on growth in *Arabidopsis* in an open system; they concluded that presence of volatiles significantly increased plant growth and a possible role of bacterially emitted CO_2_ was suggested. More recently, Minerdi et al. (2011) found that volatiles emitted from *Fusarium oxysporum*, specifically β-Caryophyllene, increased root and shoot length as well as fresh biomass of *Lactuca sativa*. They concluded that this increased growth was the result of the upregulation of seven expansin proteins.

Increase in root growth as a result of exposure to VOCs has been widely discussed in the literature (Zhang et al., 2007; Minerdi et al., 2011; Zamioudis et al., 2013) and is a likely explanation of the results in the current study. VOCs may also explain how *F. vesca* were able to locate nutrient-rich patches in past experiments (Roiloa and Retuerto, 2006). While not directly addressed in the current study, this mechanism of patch-detection might also contribute to the increased root-foraging plasticity in aggressive invaders (Keser et al., 2014), which are perhaps more sensitive or more responsive to volatile clues of nutrient availability. Our on-going studies seek to elucidate mechanisms governing plant foraging by examining the ability of individuals to respond to specific volatile cues emitted from substrates and how these volatiles might elicit specific responses in plant foraging and invasion. We want to determine whether specific growth promoting volatiles are emitted by live *versus* dead substrates and whether they affect stolon trajectory given that establishing a growth trajectory toward a nutrient-rich patch is a necessary precursor to colonization and successful nutrient foraging.

## Conflict of Interest Statement

The authors declare that the research was conducted in the absence of any commercial or financial relationships that could be construed as a potential conflict of interest.
